# BRINGING THE HAND TO THE HEAD: AGE-RELATED CHANGES IN MOVEMENT STRATEGY

**DOI:** 10.1093/geroni/igac059.2827

**Published:** 2022-12-20

**Authors:** Andrew Droste, Stephen Cain, Tanner Trenary, Rachel Logue Cook, Alanson Sample, David Burke, Paula Anne Newman-Casey, Susan Brown

**Affiliations:** University of Michigan Medicine - Kellogg Eye Center, Ann Arbor, Michigan, United States; West Virginia University, Morgantown, West Virginia, United States; University of Michigan, Ann Arbor, Michigan, United States; University of Michigan, Ann Arbor, Michigan, United States; University of Michigan, Ann Arbor, Michigan, United States; University of Michigan Medical School, Ann Arbor, Michigan, United States; University of Michigan Medicine - Kellogg Eye Center, Ann Arbor, Michigan, United States; University of Michigan, Ann Arbor, Michigan, United States

## Abstract

Functional independence in older adults relies heavily on the ability to perform daily activities requiring motion of the upper extremity that brings the hand to the head. Such tasks include hygiene activities, eating and drinking, adjusting hearing aids, or putting in eye drops – all of which can be challenging for the older individual. Despite the importance of these activities of daily living, little is known regarding age-related changes in control strategies that may contribute to performance impairments. Twelve older (mean age: 75±5.5y) and 12 young (mean age: 23±1.6y) participants performed arm movements that stopped in front of the ipsilateral eye. Movements were made under proprioceptive guidance by occluding visual feedback via blindfold. Participants first made a reference movement to determine final hand position and then reproduced the memory-based movement. The task was performed by each arm from a seated position. Inertial sensors attached to each wrist captured movement data, which were used to calculate movement characteristics (velocities, timing, smoothness). As expected, proprioceptively guided movements made by the young group were highly irregular, particularly during the deceleration phase, indicating intermittent proprioceptive monitoring of arm position in the absence of vision. In contrast, a clear difference in movement strategy was seen in older adults where movements were faster and smoother (p < 0.05), suggestive of impaired utilization of movement-related position feedback. Future studies will determine to what extent such age-related differences in control strategy contribute to functional difficulties with tasks requiring accurate placement of the hand to the head.

